# Identification of factors that promote biogenesis of tRNA_CGA_^Ser^

**DOI:** 10.1080/15476286.2018.1526539

**Published:** 2018-10-18

**Authors:** Fu Xu, Yang Zhou, Anders S. Byström, Marcus J.O. Johansson

**Affiliations:** Department of Molecular Biology, Umeå University, Umeå, Sweden

**Keywords:** *sup61*, tRNA^Ser^, tRNA maturation, tRNA modification, modified nucleosides

## Abstract

A wide variety of factors are required for the conversion of pre-tRNA molecules into the mature tRNAs that function in translation. To identify factors influencing tRNA biogenesis, we previously performed a screen for strains carrying mutations that induce lethality when combined with a *sup61-T47:2C* allele, encoding a mutant form of ${tRNA}_{CGA}^{Ser}$. Analyzes of two complementation groups led to the identification of Tan1 as a protein involved in formation of the modified nucleoside *N*^4^-acetylcytidine (ac^4^C) in tRNA and Bud13 as a factor controlling the levels of ac^4^C by promoting *TAN1* pre-mRNA splicing. Here, we describe the remaining complementation groups and show that they include strains with mutations in genes for known tRNA biogenesis factors that modify (*DUS2, MOD5* and *TRM1*), transport (*LOS1*), or aminoacylate (*SES1*) ${tRNA}_{CGA}^{Ser}$. Other strains carried mutations in genes for factors involved in rRNA/mRNA synthesis (*RPA49, RRN3* and *MOT1*) or magnesium uptake (*ALR1*). We show that mutations in not only *DUS2, LOS1* and *SES1* but also in *RPA49, RRN3* and *MOT1* cause a reduction in the levels of the altered ${tRNA}_{CGA}^{Ser}$. These results indicate that Rpa49, Rrn3 and Mot1 directly or indirectly influence ${tRNA}_{CGA}^{Ser}$ biogenesis.

## Introduction

Eukaryotic transfer RNA genes are transcribed by RNA polymerase III generating precursor tRNAs (pre-tRNAs) that undergo a series of processing steps to generate mature functional tRNAs [1–3]. These steps include the removal of 5´ leader and 3´ trailer sequences, posttranscriptional addition of CCA to the 3´ termini, and the removal of intervening sequences in pre-tRNAs transcribed from intron-containing genes. All pre-tRNAs also undergo post-transcriptional modification of a subset of their nucleosides [1–3]. Some modified nucleosides are found in essentially all tRNA species while others are present in a specific or a subclass of tRNAs. Many pre-tRNA processing steps occur in the nucleus, including end-maturation and numerous nucleoside modifications [1–3]. However, some nucleoside modifications only occur after nuclear export. Moreover, the subcellular location of pre-tRNA splicing varies between organisms and in *Saccharomyces cerevisiae* splicing occurs after the pre-tRNA has been exported to the cytoplasm.

The essential and intron-containing *sup61^+^* (*tS(CGA)C*) gene encodes the only serine isoacceptor, ${tRNA}_{CGA}^{Ser}$, that efficiently decodes UCG codons in *S. cerevisiae* [4]. We previously identified four mutant *sup61* alleles in a screen for strains that require the *TRM2* gene, encoding the tRNA (m^5^U_54_) methyltransferase, for growth [5]. The *sup61 trm2* double mutants are viable at 25°C but inviable or very slow growing at 30°C. The m^5^U_54_ residue is present in ${tRNA}_{CGA}^{Ser}$ and the lack of Trm2 causes decreased abundance of the altered ${tRNA}_{CGA}^{Ser}$ species. In addition to the requirement for *TRM2*, direct tests showed that the growth of the *sup61* mutants is dependent on other enzymes that catalyze nucleoside modifications in ${tRNA}_{CGA}^{Ser}$ [5]. These enzymes (Pus4, Trm1, and Trm3) are required for the formation of pseudouridine (Ψ) at position 55, *N*^2^,*N*^2^-dimethylguanosine ($m_{2}^{2}G$) at position 26 and 2′-O-methylguanosine (Gm) at position 18 in tRNAs [6–8]. Similar to the absence of Trm2, the lack of Pus4, Trm1, or Trm3 causes a reduction in the levels of the altered ${tRNA}_{CGA}^{Ser}$. Moreover, introduction of a null allele of *LHP1* into the *sup61* mutants was found to inhibit growth and reduce the levels of the altered ${tRNA}_{CGA}^{Ser}$ [5]. The Lhp1 protein promotes tRNA folding and protects the 3ʹ end of pre-tRNAs from exonucleolytic digestion [9,10]. Collectively, these findings suggest that the ${tRNA}_{CGA}^{Ser}$ species in the *sup61* mutants is sensitized to perturbations of the tRNA maturation pathway.

To identify novel factors promoting tRNA maturation, we performed a screen for mutations lethal in combination with one of the four *sup61* alleles [11]. The screen identified mutants representing 12 different complementation groups and detailed analyzes of one group led to the identification of *TAN1* as a gene required for the formation of *N*^4^-acetylcytidine (ac^4^C) in tRNA [11]. The ac^4^C nucleoside is present at position 12 in leucine and serine isoacceptors, and the inactivation of *TAN1* causes a lack of ac^4^C in tRNA and a reduction in the abundance of the altered ${tRNA}_{CGA}^{Ser}$ [11]. Another complementation group consisted of a strain with a mutation in the *BUD13* gene [12]. *BUD13* encodes a subunit of the heterotrimeric pre-mRNA retention and splicing (RES) complex, which promotes splicing and nuclear retention of a subset of intron-containing pre-mRNAs [13–18]. The requirement for Bud13 in the *sup61* mutant is caused by an important role of the RES complex in splicing and nuclear retention of *TAN1* pre-mRNA and consequently in the formation of ac^4^C [12]. In this study, we describe the remaining mutants identified in the screen and show that they do not only define strains with mutations in genes for known tRNA biogenesis factors but also those involved in RNA polymerase I and II transcription or Mg^2+^ uptake.

## Results

### Several different factors that promote modification of $tRNA_{CGA}^{Ser}$ are required for growth of *sup61-T47:2C* cells

**Figure 1.**
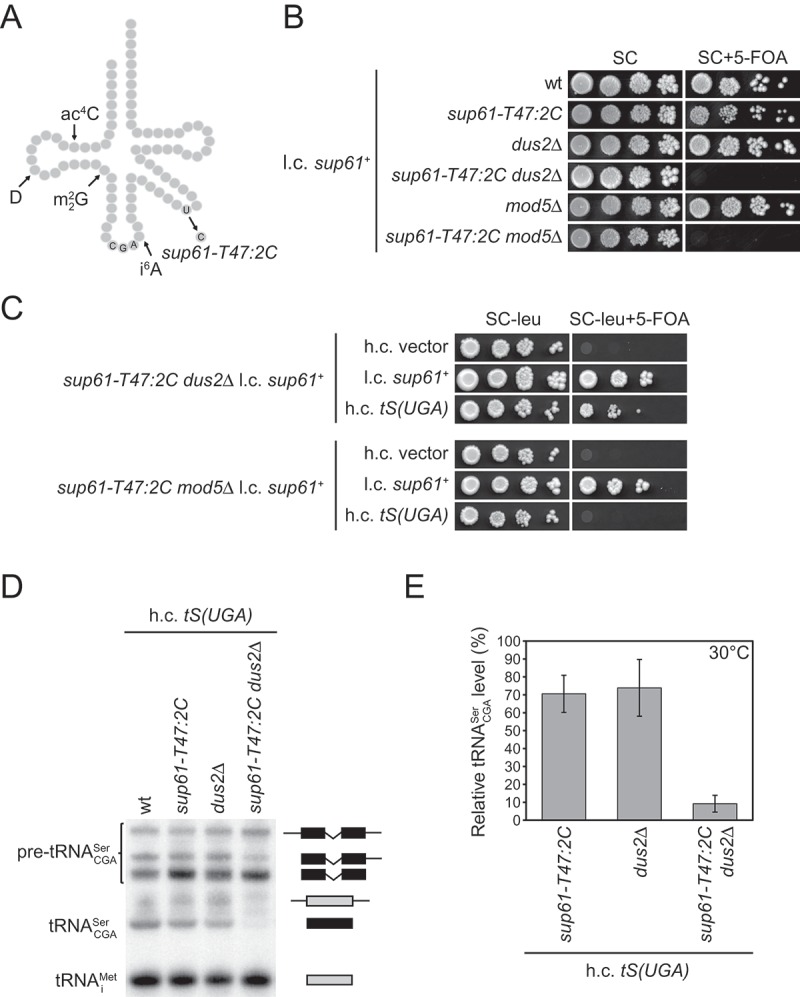
The *DUS2* and *MOD5* genes are required for growth of *sup61-T47:2C* cells. (a) Schematic secondary structure of ${tRNA}_{CGA}^{Ser}$. The alteration caused by the *sup61-T47:2C* allele and the positions of ac^4^C_12_, D_20_, $m_{2}^{2}G$_26_ and i^6^A_37_ are indicated. (b) Effects of *dus2Δ* and *mod5Δ* alleles on growth of *sup61-T47:2C* cells. The wild-type (UMY2220), *sup61-T47:2C* (MJY926), *dus2Δ (*UMY2872), *sup61-T47:2C dus2Δ* (UMY4286), *mod5Δ* (UMY2565), and *sup61-T47:2C mod5Δ* (UMY4285) strains carrying the low-copy (l.c.) *URA3* plasmid pRS316-*sup61^+^* were grown over-night at 30°C in liquid synthetic complete medium (SC). The cells were serially diluted, spotted onto SC plates and SC plates containing 5-fluoroorotic acid (SC+ 5-FOA), and incubated at 30°C for 3 days. Only cells that have lost the *URA3* plasmid are able to grow on 5-FOA-containing medium [64]. (c) Effects of increased *tS(UGA)P* dosage on growth of *sup61-T47:2C dus2Δ* and *sup61-T47:2C mod5Δ* cells. The *sup61-T47:2C dus2Δ* and *sup61-T47:2C mod5Δ* strains from (b) were transformed with the empty high copy (h.c) *LEU2* plasmid pRS425 [65], the l.c. *LEU2* plasmid pRS315-*sup61^+^*, and the h.c. *LEU2* plasmid pRS425-*tS(UGA)P*. After purifications by single cell streaks on SC plates lacking uracil and leucine (SC-ura-leu), the transformants were grown over-night at 30°C in liquid SC medium lacking leucine (SC-leu). Cells were serially diluted, spotted onto SC-leu and SC-leu+ 5-FOA plates, and incubated at 30°C for 3 days. (d) Northern analysis of total RNA isolated from the wild-type, *sup61-T47:2C, dus2*Δ, and *sup61-T47:2C dus2*Δ strains carrying the h.c. *LEU2* plasmid pRS425-*tS(UGA)P*. The cells were grown in SC-leu medium at 30°C. The blot was probed for pre-${tRNA}_{CGA}^{Ser}$, ${tRNA}_{CGA}^{Ser}$, and ${tRNA}_{i}^{Met}$ using radiolabeled oligonucleotides. ${tRNA}_{i}^{Met}$ serves as the loading control. The position and identity of the ${tRNA}_{CGA}^{Ser}$ (black) and ${tRNA}_{i}^{Met}$ (gray) species are indicated on the right. (e) Influence of a *dus2Δ* allele on the abundance of the mature ${tRNA}_{CGA}^{Ser}$. The mature ${tRNA}_{CGA}^{Ser}$ signal in (d) and two additional independent experiments was normalized to the corresponding ${tRNA}_{i}^{Met}$ signal and the value for each strain expressed relative to that for the wild-type, which was set to 100%. The standard deviation is indicated.

The screen for mutations lethal in combination with the *sup61-T47:2C* allele, encoding a ${tRNA}_{CGA}^{Ser}$ species with an alteration in the variable arm (Figure 1(a)), identified mutants representing 12 complementation groups [11]. One group consisted of strains in which the mutation was genetically linked to the *sup61* locus and this group was excluded from further analysis. The mutant gene in each of the 11 remaining groups (Table 1) was identified by complementing the phenotype with a yeast genomic library and subsequent confirmation that the original mutation was genetically linked to the locus for the complementing gene. As the *sup61-T47:2C* allele generates a requirement for several different tRNA modifying enzymes [5,11], we expected that the screen would identify factors involved in the modification of ${tRNA}_{CGA}^{Ser}$. In addition to *trm1, tan1* and *bud13* mutants, the screen identified strains with mutations in the *DUS2* and *MOD5* genes (Table 1) [11,12,19]. These genes encode the tRNA modifying enzymes that catalyze the formation of dihydrouridine (D) at position 20 and *N*^6^-isopentenyladenosine (i^6^A) at position 37, respectively (Figure 1(a)) [20–23]. To demonstrate unambiguously that *DUS2* and *MOD5* are required for growth of cells with the altered ${tRNA}_{CGA}^{Ser}$, we independently combined, in the presence of a rescuing *sup61^+^*-containing plasmid, the *sup61-T47:2C* mutation with *dus2Δ* and *mod5Δ* alleles. The resulting *sup61-T47:2C dus2Δ* and *sup61-T47:2C mod5Δ* strains were dependent on the plasmid for growth at both 30°C and 25°C (Figures 1(b) and S1), confirming that the alteration in ${tRNA}_{CGA}^{Ser}$ causes a requirement for Dus2 and Mod5.

**Table 1.** The mutants define twelve complementation groups.

Complementation group	Number of mutants	Gene
I	3	*sup61-T47:2C*
II	2	*TRM1*
III	3	*TAN1*
IV	1	*BUD13*
V	2	*DUS2*
VI	2	*MOD5*
VII	2	*SES1*
VIII	1	*LOS1*
IX	1	*MOT1*
X	2	*RPA49*
XI	1	*RRN3*
XII	1	*ALR1*

As the inactivation of any of several tRNA modifying factors in *sup61-T47:2C* cells causes a decreased abundance of the altered ${tRNA}_{CGA}^{Ser}$ [5,11], it seemed likely that mutations in *DUS2* or *MOD5* would induce a similar defect. Even though the *sup61^+^* gene is essential, we previously showed that the inviability of *sup61Δ* cells is suppressed by increased dosage of a gene (*tS(UGA)P*) coding for ${tRNA}_{ncm5UGA}^{Ser}$ [24]. This finding suggested that the introduction of a high-copy *tS(UGA)P* plasmid into the double mutants may suppress the dependence on the *sup61^+^* plasmid and allow analyzes of ${tRNA}_{CGA}^{Ser}$ levels. Increased dosage of the *tS(UGA)P* gene counteracted the requirement for the *sup61^+^* plasmid in the *sup61-T47:2C dus2Δ* strain both at 30°C and 25°C (Figures 1(c) and S1). However, the requirement for the *sup61^+^* plasmid was not suppressed by increased *tS(UGA)P* dosage in *sup61-T47:2C mod5Δ* cells, preventing further analyzes of the strain (Figures 1(c) and S1). An i^6^A_37_ residue is also found in ${tRNA}_{ncm5UGA}^{Ser}$ [25] and the inability of the high-copy *tS(UGA)P* plasmid to suppress the *sup61-T47:2C mod5Δ* strain suggests that the isopentenyl group at A_37_ enhances the ability of ${tRNA}_{ncm5UGA}^{Ser}$ to read the near-cognate UCG codon.

To investigate the influence of Dus2 on ${tRNA}_{CGA}^{Ser}$ abundance, we used northern blotting to analyze ${tRNA}_{CGA}^{Ser}$ transcripts in wild-type, *sup61-T47:2C, dus2Δ*, and *sup61-T47:2C dus2Δ* strains carrying the high-copy *tS(UGA)P* plasmid. The blots were also probed for ${tRNA}_{i}^{Met}$, which served as the loading control. The analyzes revealed a decreased abundance of mature ${tRNA}_{CGA}^{Ser}$ in the *dus2Δ sup61-T47:2C* strain compared to the *dus2Δ* and *sup61-T47:2C* single mutants (Figure 1(d,e)). Thus, the effect of the *dus2Δ* allele on the abundance of the altered ${tRNA}_{CGA}^{Ser}$ is similar to that observed for the inactivation of other tRNA modifying factors [5,11].

### The abundance of the *sup61-T47:2C*-encoded $tRNA_{CGA}^{Ser}$ is reduced in cells with mutant *ses1* or *los1* alleles

**Figure 2.**
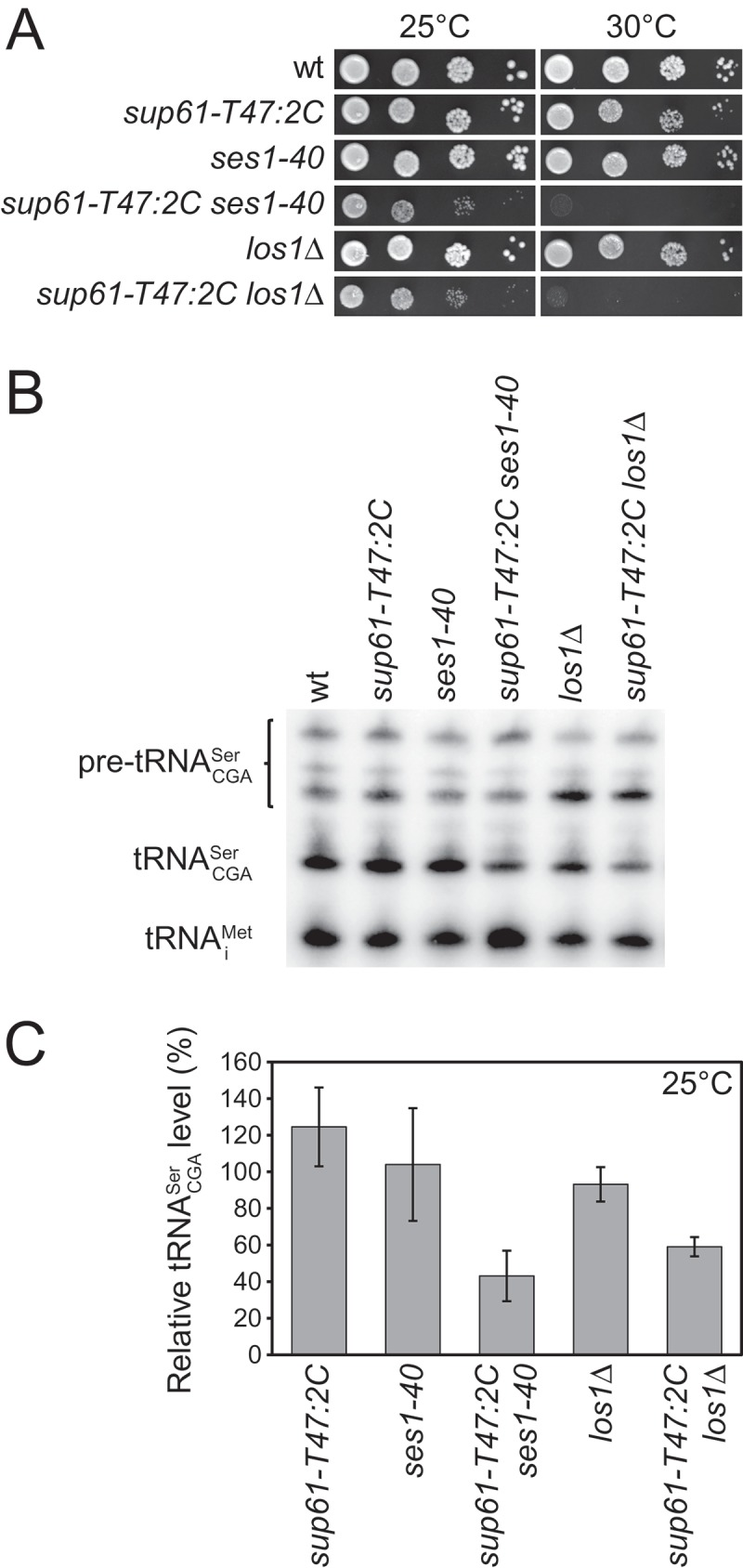
Influence of the *ses1-40 and los1Δ* alleles on growth and ${tRNA}_{CGA}^{Ser}$abundance. (a) Effects of the *ses1-40 and los1Δ* alleles on growth of *sup61-T47:2C* cells. The wild-type (UMY2220), *sup61-T47:2C* (MJY926), *ses1-40* (MJY924), *sup61-T47:2C ses1-40* (MJY925), *los1Δ (*UMY2441), and *sup61-T47:2C los1Δ* (UMY2861) strains were grown over-night at 25°C in liquid SC medium. The cells were serially diluted, spotted onto SC plates, and incubated at 25°C for 3 or at 30°C for 2 days. (b) Northern analysis of total RNA isolated from the strains described in (a) grown in SC medium at 25°C. The blot was probed for pre-${tRNA}_{CGA}^{Ser}$, ${tRNA}_{CGA}^{Ser}$, and ${tRNA}_{i}^{Met}$ using radiolabeled oligonucleotides. ${tRNA}_{i}^{Met}$ serves as the loading control. (c) Influence of *ses1-40 and los1Δ* alleles on the levels of mature ${tRNA}_{CGA}^{Ser}$. The ${tRNA}_{CGA}^{Ser}$ signal in (b) and two additional independent experiments was normalized to the corresponding ${tRNA}_{i}^{Met}$ signal and the value for each strain expressed relative to that for the wild-type, which was set to 100%. The standard deviation is indicated.

In addition to tRNA modification mutants, we expected that the screen would define strains with reduced function in other steps of tRNA biogenesis. Accordingly, two of the remaining complementation groups consisted of strains carrying mutations in the *SES1* or *LOS1* gene (Table 1). The essential *SES1* gene codes for the seryl-tRNA synthetase that aminoacylates serine tRNA isoacceptors [26] whereas *LOS1* encodes a nonessential factor that mediates nuclear export of pre-tRNAs [3,27–29]. One of the two isolated *sup61-T47:2C ses1* double mutants *(ses1-40)* was able to lose the rescuing *sup61^+^* vector at 25°C, generating a strain that is slow-growing at 25°C and inviable at 30°C (Figure 2(a)). Similarly, a *sup61-T47:2C los1Δ* double mutant is slow-growing at 25°C and inviable at 30°C (Figure 2(a)). Northern blot analyzes of wild-type, *sup61-T47:2C, ses1-40, sup61-T47:2C ses1-40, los1Δ*, and *sup61-T47:2C los1Δ* cells grown at 25°C revealed that the abundance of the mature ${tRNA}_{CGA}^{Ser}$ is reduced in the double mutants compared to the respective single mutants (Figure 2(b,c)). Further, the *ses1-40 sup61-T47:2C* mutant shows reduced levels of partially processed pre-${tRNA}_{CGA}^{Ser}$ (Figure 2(b) and Table S1), indicating that the stability and/or processing of the mutant pre-${tRNA}_{CGA}^{Ser}$ is affected by the *ses1-40* allele. Similarly, the accumulation of unspliced pre-${tRNA}_{CGA}^{Ser}$ in *los1Δ* cells [27,30] is less pronounced in the *sup61-T47:2C los1Δ* mutant (Figure 2(b) and Table S1).

In addition to its essential role in aminoacylation, Ses1 was recently shown to stimulate the formation of 3-methylcytidine (m^3^C) at position 32 in ${tRNA}_{CGA}^{Ser}$ and ${tRNA}_{ncm5UGA}^{Ser}$ [31]. The methylation of C_32_ is catalyzed by the Trm140 protein and the modification is known to be present in three out of four tRNA^Ser^ and all three tRNA^Thr^ species [32,33]. Ses1 was found to co-purify with Trm140 and its stimulatory role in the formation of m^3^C was restricted to the serine isoacceptors [31]. To investigate the possibility that the *ses1-40* allele influences the viability of *sup61-T47:2C* cells by inducing a tRNA modification defect, we used HPLC to analyze the nucleoside composition of total tRNA from wild-type and *ses1-40* cells grown at 30°C. We also analyzed the nucleoside composition of total tRNA from the *los1Δ* mutant grown at 30°C. The analyzes showed that the levels of the modified nucleosides normally found in ${tRNA}_{CGA}^{Ser}$, including m^3^C, are unaffected in total tRNA from the *ses1-40* and *los1Δ* mutants (Table S2 and Figure S2). However, any effect of the *ses1-40* allele on the m^3^C levels in the serine isoacceptors may be masked by the m^3^C residues in the tRNA^Thr^ species. To directly test if the absence of m^3^C affects growth of *sup61-T47:2C* cells, we combined a *trm140Δ* allele with the *sup61-T47:2C* mutation. The *sup61-T47:2C trm140Δ* strain was viable with only a minor growth defect compared to the *sup61-T47:2C* single mutant (Figure S2). This finding suggests that any effect of the *ses1-40* allele on the formation of m^3^C in ${tRNA}_{CGA}^{Ser}$ is not the cause of the growth defect of *sup61-T47:2C ses1-40* cells. Collectively, our results show that Ses1 and Los1 are important to maintain the levels of the altered ${tRNA}_{CGA}^{Ser}$.

### Factors involved in RNA polymerase I and II transcription influence the growth of *sup61-T47:2C* cells

Three complementation groups defined strains with mutations in genes, *RPA49, RRN3* or *MOT1*, encoding factors involved in RNA polymerase I and/or II transcription (Table 1). *RPA49* encodes a nonessential subunit of RNA polymerase I (Pol I) [34] whereas *RRN3* codes for an essential Pol I transcription factor [35]. The *MOT1* gene codes for an essential protein that has global effects on gene expression by controlling the distribution and activity of the TATA-binding protein (TBP) at promoters of RNA polymerase II (Pol II) transcribed genes [36–40]. In addition to its role in Pol II transcription, Mot1 has also been suggested to regulate Pol I transcription [41].

**Figure 3.**
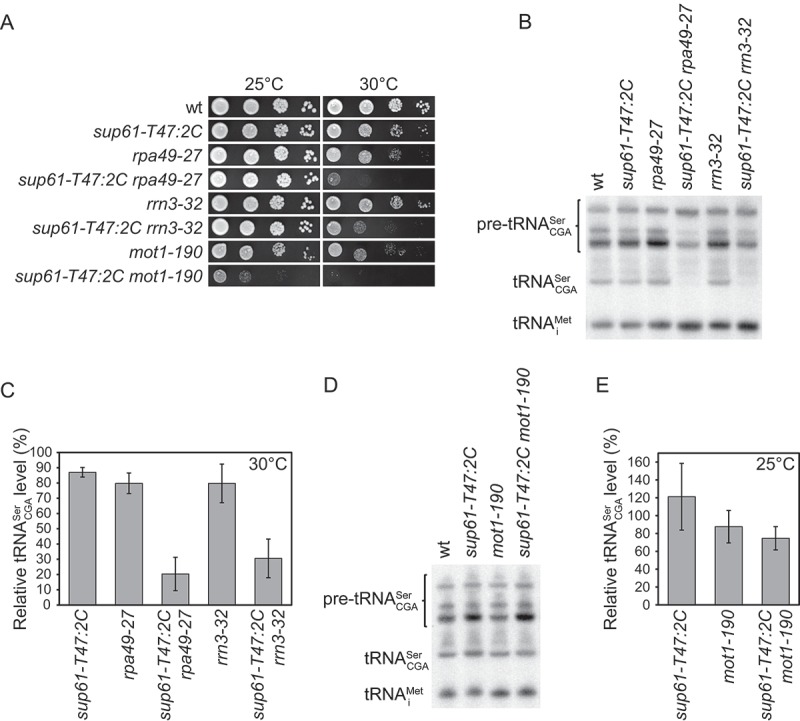
Mutations in the *MOT1, RPA49* and *RRN3* genes are detrimental to cells with a *sup61-T47:2C* allele. (a) Effects of the *mot1-190, rpa49-27 and rrn3-32* alleles on growth of *sup61-T47:2C* cells. The wild-type (UMY2220), *sup61-T47:2C* (MJY926), *rpa49-27* (MJY890), *sup61-T47:2C rpa49-27* (MJY891), *rrn3-32* (MJY894), *sup61-T47:2C rrn3-32* (MJY895), *mot1-190* (MJY892), and *sup61-T47:2C mot1-190* (MJY893) strains were grown over-night at 25°C in liquid SC medium. Cells were serially diluted, spotted onto SC plates, and incubated at 25°C for 3 or at 30°C for 2 days. (b) Northern blot analysis of total RNA isolated from wild-type, *sup61-T47:2C, rpa49-27, sup61-T47:2C rpa49-27, rrn3-32* and *sup61-T47:2C rrn3-32* strains grown in SC medium at 30°C. The blot was probed for pre-${tRNA}_{CGA}^{Ser}$, ${tRNA}_{CGA}^{Ser}$, and ${tRNA}_{i}^{Met}$ using radiolabeled oligonucleotides.${tRNA}_{i}^{Met}$ serves as the loading control. (c) Influence of *rpa49-27 and rrn3-32* alleles on the levels of mature ${tRNA}_{CGA}^{Ser}$. The ${tRNA}_{CGA}^{Ser}$ signal in (b) and two additional independent experiments was normalized to the corresponding ${tRNA}_{i}^{Met}$ signal and the value for each strain expressed relative to that for the wild-type, which was set to 100%. The standard deviation is indicated. (d) Northern blot analysis of total RNA isolated from wild-type, *sup61-T47:2C, mot1-190*, and *sup61-T47:2C mot1-190* strains grown in SC medium at 25°C. The blot was probed as described in (b). (e) Influence of the *mot1-190* allele on ${tRNA}_{CGA}^{Ser}$ abundance. The ${tRNA}_{CGA}^{Ser}$ signal in (d) and three additional independent experiments was normalized to the corresponding ${tRNA}_{i}^{Met}$ signal and the value for each strain expressed relative to that for the wild-type, which was set to 100%. The standard deviation is indicated.

Cells deleted for the *RPA49* gene are viable, but they show a slow-growth phenotype that is more pronounced at 25°C than at 30°C (Figure S3) [34,42]. However, the growth defect caused by one of the two *rpa49* alleles (*rpa49-27*) identified from the screen was less severe than that induced by the deletion (Figure S3). The *sup61-T47:2C rpa49-27* strain was able to lose the rescuing *sup61^+^* plasmid at 25°C generating a strain that is viable but very slow growing at 30°C (Figure 3(a)). Similarly, the *sup61-T47:2C rrn3-32* and *sup61-T47:2C mot1-190* strains were able to lose the rescuing *sup61^+^* plasmid at 25°C. The *sup61-T47:2C rrn3-32* strain is viable but slow-growing at 30°C whereas the *sup61-T47:2C mot1-190* strain is inviable at 30°C and very slow-growing at 25°C (Figure 3(a)).

The effect of the *rpa49-27, rrn3-32* and *mot1-190* alleles on ${tRNA}_{CGA}^{Ser}$abundance was examined in cells grown at either 30°C (*rrn3-32* and *rpa49-27*) or 25°C (*mot1-190*). These analyzes revealed that the level of the mature ${tRNA}_{CGA}^{Ser}$ is reduced in the *sup61-T47:2C rrn3-32* and *sup61-T47:2C rpa49-27* strains compared to the respective single mutants (Figure 3(b,c)). Moreover, the *sup61-T47:2C rrn3-32* and *sup61-T47:2C rpa49-27* strains show reduced levels of partially processed pre-${tRNA}_{CGA}^{Ser}$ (Figure 3(b) and Table S1). Only a slight decrease in ${tRNA}_{CGA}^{Ser}$ levels was observed in the *sup61-T47:2C mot1-190* strain (Figure 3(d,e)), suggesting that the effect on ${tRNA}_{CGA}^{Ser}$ abundance may not, at least by itself, explain the severe growth defect of the double mutant. Analyzes of the nucleoside composition of total tRNA from the *rpa49-27, rrn3-32* and *mot1-190* single mutants revealed no apparent tRNA modification defect (Table S2), indicating that Rpa49, Rrn3 and Mot1 influences ${tRNA}_{CGA}^{Ser}$ levels by another mechanism.

### Mutations in the *ALR1* gene are detrimental for growth of *sup61-T47:2C* cells

**Figure 4.**
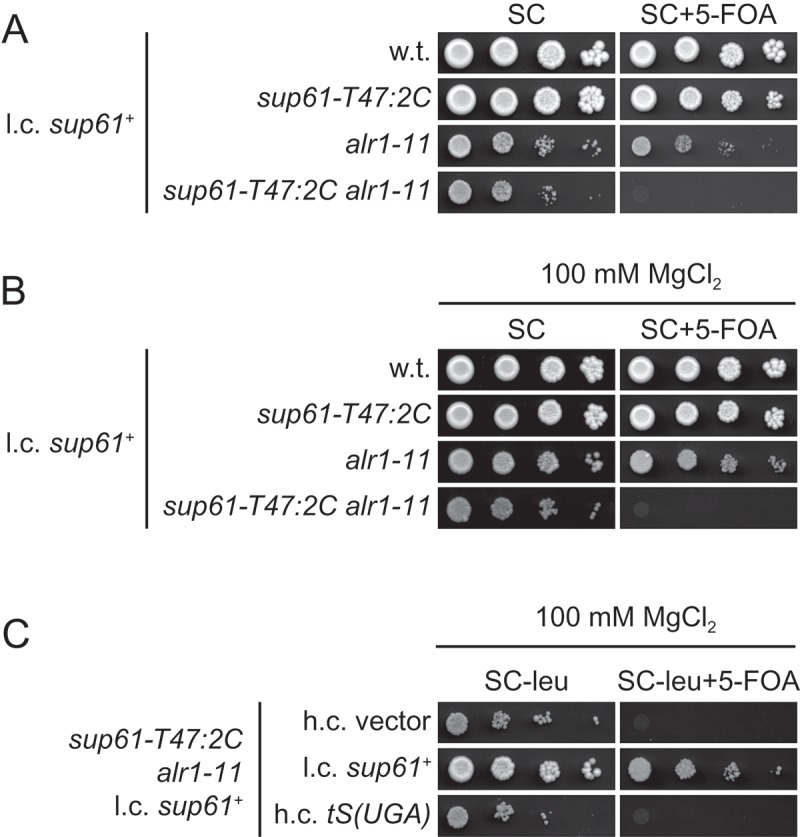
Genetic interactions between the *alr1-11* and *sup61-T47:2C* alleles. (a and b) Growth phenotypes of strains with an *alr1-11* and/or *sup61-T47:2C* allele. The wild-type (UMY2220), *sup61-T47:2C* (MJY926), *alr1-11* (UMY3002), *sup61-T47:2C alr1-11* (UMY2967) strains carrying the l.c. *URA3* plasmid pMJ1421 (*sup61^+^*) were grown overnight at 30°C in liquid SC medium (A) or in liquid SC medium supplemented with 100 mM MgCl_2_ (b). Cells were serially diluted, spotted onto SC and SC+ 5-FOA (A) or SC and SC+ 5-FOA plates containing 100 mM MgCl_2_ (b), and incubated at 30°C for 3 days. (c) Effect of increased *tS(UGA)P* dosage on growth of *sup61-T47:2C alr1-11* cells. The *sup61-T47:2C alr1-11* strain described in (a) was transformed with the indicated h.c. or l.c. *LEU2* plasmids. Transformants were grown at 30°C in liquid SC-leu medium supplemented with 100 mM MgCl_2_. Cells were serially diluted, spotted onto SC-leu and SC-leu+ 5-FOA plates containing 100 mM MgCl_2_, and incubated at 30°C for 3 days.

The final complementation group consisted of a strain with a mutation in the *ALR1* gene, which encodes the main Mg^2+^ importer of *S. cerevisiae* [43,44]. In addition to the importance of Mg^2+^ as a cofactor/counterion, it plays an important role in establishing and maintaining structures of nucleic acids, proteins and membranes. Strains with mutations in *ALR1* show reduced intracellular Mg^2+^ levels and an *alr1Δ* mutant is, depending on genetic background, either inviable or very slow growing on standard media [43–45]. These phenotypes are partially suppressed by supplementation of the media with Mg^2+^ [43,44]. We found that supplementation of the medium with Mg^2+^ does not suppress the *sup61^+^*-plasmid dependence of *sup61-T47:2C alr1-11* cells at either 30°C or 25°C (Figures 4(a, b) and S4). Moreover, analyzes of the effects of the *alr1* allele on ${tRNA}_{CGA}^{Ser}$ levels was prevented by the finding that increased ${tRNA}_{ncm5UGA}^{Ser}$ expression does not suppress the plasmid dependence of the *sup61-T47:2C alr1-11* strain even on Mg^2+^-supplemented medium (Figures 4(c) and S4). As no tRNA modification defect is apparent in *alr1-11* cells (Table S2), the cause of the synergistic interaction remains unclear. Nevertheless, our findings suggest that cells with the altered ${tRNA}_{CGA}^{Ser}$ are sensitive to defects in Mg^2+^ uptake.

## Discussion

We previously showed that the viability of *sup61-T47:2C* cells is dependent on several different factors promoting maturation of ${tRNA}_{CGA}^{Ser}$ [5,11,12]. In this study, we describe additional factors required for growth of strains with the altered form of ${tRNA}_{CGA}^{Ser}$. We show that mutations in genes required for modification (*DUS2* and *MOD5*), transport (*LOS1*), and aminoacylation (*SES1*) of ${tRNA}_{CGA}^{Ser}$ are deleterious to cells with the *sup61-T47:2C* allele. Similar to the inactivation of other factors influencing tRNA biogenesis, the *dus2, los1*, and *ses1* alleles cause a reduced abundance of the altered ${tRNA}_{CGA}^{Ser}$. These findings provide further support for the notion that *sup61-T47:2C* cells are sensitized for perturbations in tRNA biogenesis.

In addition to mutations in genes for known tRNA biogenesis factors, we found that mutations in the *MOT1, RPA49*, or *RRN3* gene inhibit growth of *sup61-T47:2C* cells and cause a reduction in the levels of the altered ${tRNA}_{CGA}^{Ser}$. The Mot1 protein is thought to influence both Pol I and Pol II transcription [36–41,46] whereas the function of Rpa49 and Rrn3 appears to be restricted to Pol I transcription [34,35,42]. The mechanisms by which Mot1, Rpa49 and Rrn3 influence tRNA biogenesis is unclear, but it is possible that they indirectly influence the process through their involvement in Pol I transcription. Transcription of tRNA genes as well as 5´-end processing of pre-tRNAs has been reported to localize to the nucleolus [47–49]. Moreover, dissociation of pre-tRNA from the nucleolus and defects in 5´ end processing have been observed in cells defective in Pol I transcription or rRNA processing [50,51]. As mutations in *MOT1, RPA49*, and *RRN3* affect nucleolar morphology [52,53], the *mot1-190, rpa49-27*, and *rrn3-32* alleles may influence ${tRNA}_{CGA}^{Ser}$ biogenesis by disrupting the balance and/or order of pre-tRNA processing. The *rpa49-27* and *rrn3-32* alleles induce reduced levels of not only the mature but also the partially processed forms of the altered ${tRNA}_{CGA}^{Ser}$ (Figure 3(b) and Table S1), indicating that pre-tRNA processing and/or stability is affected in the mutants. The abundance of the primary unprocessed pre-${tRNA}_{CGA}^{Ser}$ is, however, largely unaffected by the *mot1-190, rpa49-27* and *rrn3-32* alleles (Figure 3(b,d) and Table S1), suggesting that the mutations do not cause a defect in 5´-end processing.

The finding that a mutation in the *ALR1* gene is lethal in combination with the *sup61-T47:2C* allele indicates that a reduction in intracellular Mg^2+^ levels may cause inefficient folding and/or decreased stability of the altered ${tRNA}_{CGA}^{Ser}$. Alternatively, the genetic interaction could be a consequence of the effect of reduced Mg^2+^ on translational fidelity [54–56], i.e. decreased intracellular Mg^2+^ levels may reduce the ability of the altered ${tRNA}_{CGA}^{Ser}$ to read the UCG codon. It should, however, be noted that Mg^2+^ is important for a vast number of cellular processes and it cannot be excluded that the genetic interaction is caused by a more indirect mechanism.

Cells with mutations in the single-copy and essential *sup61^+^* are sensitized for defects in tRNA biogenesis. The screen for mutations lethal when combined with a *sup61-T47:2C* allele is an unbiased genetic approach to identify factors promoting tRNA biogenesis. As our screen was far from being saturated, additional factors would likely be identified in an expanded screen.

## Materials and methods

### Yeast strains, media, and genetic procedures

Strains used in this study are listed in Table S3. Yeast media were prepared essentially as described [57] using a slightly different composition of the drop-out mix [58]. Difco yeast nitrogen base without amino acids was purchased from Becton Dickinson (291940).

The screen for mutations lethal in combination with the *sup61-T47:2C* mutation and the subsequent identification of plasmids that complement the phenotype has been described [11]. To investigate linkage between the complementing gene and the original mutation, we cloned the gene or a DNA fragment close to the gene into an integrative vector and targeted the plasmid marker (*TRP1* or *URA3*) to the corresponding locus in strain UMY2256. This strain was crossed to the relevant mutant(s), carrying pMJ1421 (*URA3, ADE3, sup61^+^*) or pMJ1422 (*TRP1, ADE3, sup61^+^*) [11], and tetrad analysis revealed that the ability to lose the *sup61^+^* plasmid always co-segregated with the integrated *TRP1/URA3* marker, i.e., the mutation was in all cases linked to the locus for the complementing gene.

One copy of the *DUS2, MOD5, RPA49*, and *TRM140* genes was independently deleted in the diploid strain UMY2366 by a PCR-mediated strategy [59,60]. The generated heterozygous diploids were allowed to sporulate and the *dus2Δ* (UMY2872), *mod5Δ* (UMY2565), *rpa49Δ* (MJY1111) and *trm140Δ* (MJY1110) strains obtained from tetrads. The *dus2Δ* and *mod5Δ* strains were crossed to a *sup61-T47:2C* strain, carrying a *sup61^+^* gene on a plasmid, and rescued double mutants obtained from tetrads. The *sup61-T47:2C trm140Δ* double mutant was obtained from a cross between UMY2256 and MJY1110.

The *sup61-T47:2C alr1-11, sup61-T47:2C ses1-40, sup61-T47:2C rrn3-32, sup61-T47:2C mot1-190* and *sup61-T47:2C rpa49-27* mutants identified in the screen, all carrying a rescuing *sup61^+^*-containing plasmid, were backcrossed at least three times to UMY2256. The backcrossed strains were crossed to UMY2219 and *alr1-11, ses1-40, rrn3-32, mot1-190*, and *rpa49-27* single mutants obtained from tetrads.

### Plasmid constructions

A low copy *LEU2* plasmid harboring the *sup61^+^* gene (pRS315-*sup61^+^*) was constructed by cloning a BamHI/HindIII DNA fragment from pRS316-*sup61^+^* [5] into the corresponding sites of pRS315 [61]. Plasmids pMJ1421, pMJ1422, and pRS425-*tS(UGA)P* have been described [5,24].

### RNA methods

Total RNA for northern blotting was isolated [58] from exponentially growing cells at an optical density at 600 nm (OD_600_) of ≈ 0.5. An aliquot of slow-growing double mutants was, during harvesting, streaked on a SC plate and incubated at the appropriate temperature. Only cell pellets from cultures that contained no or negligible amounts of faster growing revertants were processed further. The procedures for northern blotting have been described [5,11]. The oligonucleotides used to detect precursor and mature ${tRNA}_{CGA}^{Ser}$ were 5´- AGCCGAACTTTTTATTCCATTCG-3´ and 5´-GCCCAAGAGATTTCGAGTCTCT-3´, respectively. To detect ${tRNA}_{i}^{Met}$, the oligonucleotide 5´-GGACATCAGGGTTATGAGCC-3´ was used.

For HPLC analyzes of modified nucleosides, the strains were grown in SC medium at 30°C to OD_600_ ≈ 0.8. Cells were harvested and total tRNA isolated as previously described [62]. Typically, 50µg of total tRNA was digested to nucleosides [63] using nuclease P1 (Sigma-Aldrich, N8630) and bacterial alkaline phosphatase (Sigma-Aldrich, P4252). The hydrolysate was analyzed by HPLC [63] using a Develosil C30 reversed phase column (Phenomenex, CH0-5690) and buffer A (0.01M NH_4_H_2_PO_4_, 2.5% methanol (v/v) at pH 5.3), buffer B (0.01M NH_4_H_2_PO_4_, 20% methanol (v/v) at pH 5.1), and buffer C (0.01M NH_4_H_2_PO_4_, 35% acetonitrile (v/v)) as the elution buffers. To be able to separate m^3^C from U, the methanol concentration in buffer A was changed to 6% (v/v).
