# Supplementary material for: Identification of factors that promote biogenesis of tRNACGASer
Source: RNA Biol. 2018 Oct 18;15(10):1286–94. doi: 10.1080/15476286.2018.1526539 (PMC6284589; doi:10.1080/15476286.2018.1526539)
Supplement: Supplemental Material [file krnb-15-10-1526539-s001.zip › Supplementary material/Supplementary Figure S1.pdf]

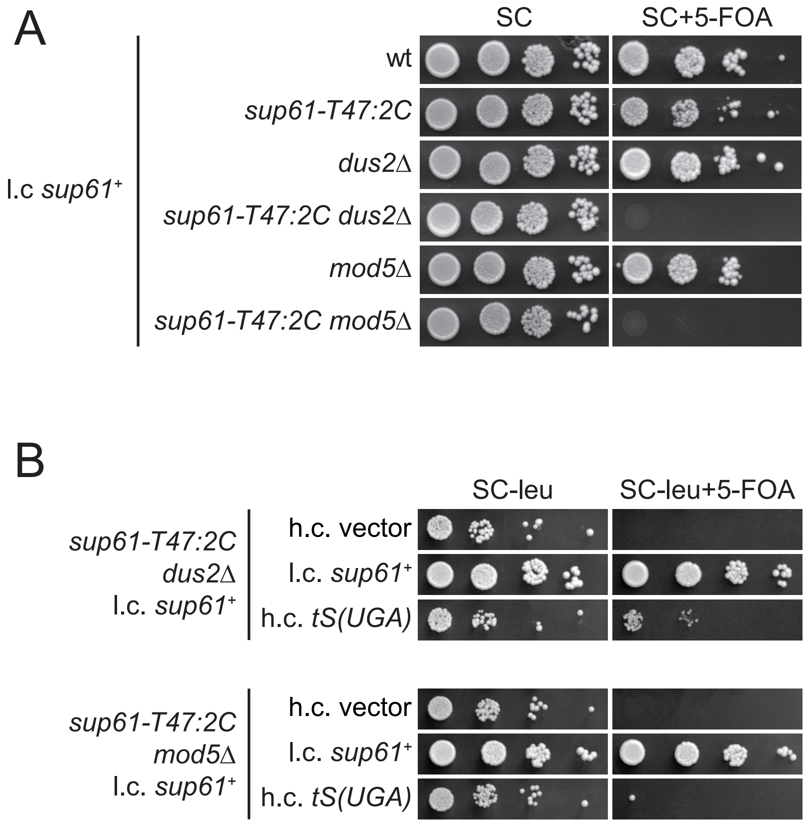

Figure S1. The *DUS2* and *MOD5* genes are required for growth of *sup61-T47:2C* cells at 25°C. (A) Effects of *dus2Δ* and *mod5Δ* alleles on growth of *sup61-T47:2C* cells. The wild-type (UMY2220), *sup61-T47:2C* (MJY926), *dus2Δ* (UMY2872), *sup61-T47:2C dus2Δ* (UMY4286), *mod5Δ* (UMY2565), and *sup61-T47:2C mod5Δ* (UMY4285) strains carrying pRS316-*sup61*+ (*URA3*) were grown over-night at 25°C in SC medium. Cells were serially diluted, spotted onto SC and SC+5-FOA plates, and incubated at 25°C for 3 days. (B) Effects of increased *tS(UGA)P* dosage on growth of *sup61-T47:2C dus2Δ* and *sup61-T47:2C mod5Δ* cells. The *sup61-T47:2C dus2Δ* and *sup61-T47:2C mod5Δ* strains from (A) were transformed with the indicated l.c. and h.c. *LEU2* plasmids. The cells were grown over-night at 25°C in liquid SC-leu medium, serially diluted, spotted onto SC-leu and SC-leu+5-FOA plates, and incubated at 25°C for 3 days.
