# Supplementary material for: Identification of factors that promote biogenesis of tRNACGASer
Source: RNA Biol. 2018 Oct 18;15(10):1286–94. doi: 10.1080/15476286.2018.1526539 (PMC6284589; doi:10.1080/15476286.2018.1526539)
Supplement: Supplemental Material [file krnb-15-10-1526539-s001.zip › Supplementary material/Supplementary Figure S2.pdf]

A

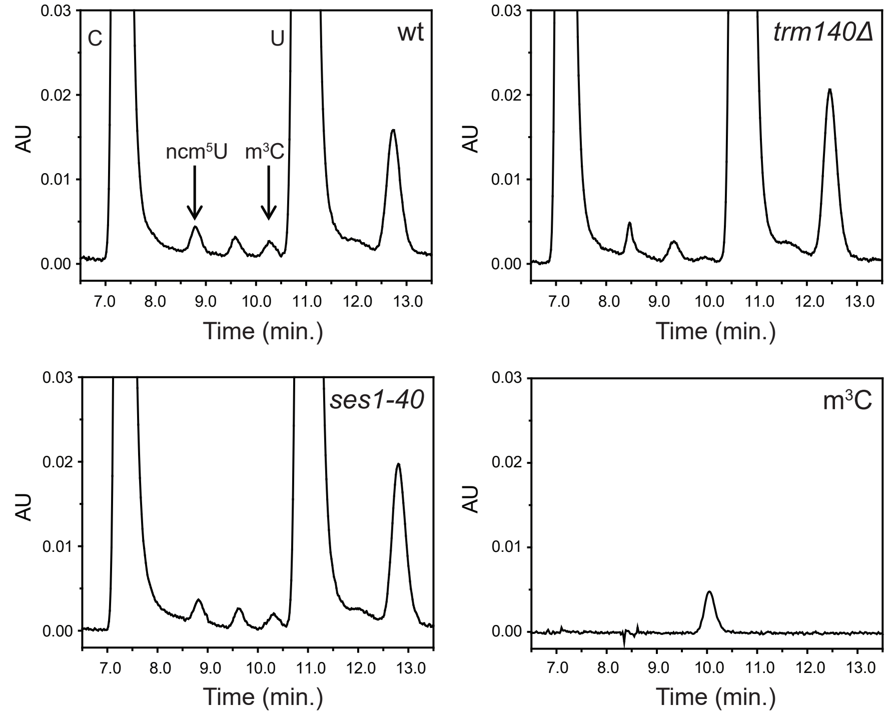

B

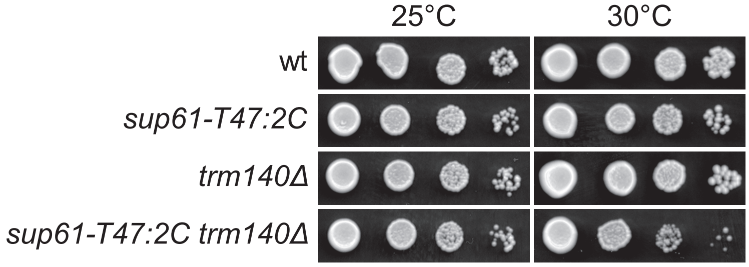

Figure S2. The influence of the *ses1-40* allele on growth of *sup61-T47:2C* cells is not a consequence of an effect on the levels of m^{3}C. (A) HPLC analyses of the nucleoside composition of total tRNA from wild-type (UMY2220), *ses1-40* (MJY924) and *trm140 Δ* (MJY1110) cells. The analysis of m^{3}C (0.3 nmole) is shown as a control. The portion of the chromatograms between 6.5 and 13.5 minutes are shown. (B) Growth phenotypes of strains with a *sup61-T47:2C* and/or *trm140*Δ allele. The wild-type (UMY2220), *sup61-T47:2C* (MJY926), *trm140Δ* (MJY1110), and *sup61-T47:2C trm140Δ* (MJY1134) strains were grown overnight at 25°C in liquid SC medium. Cells were serially diluted, spotted onto SC plates, and incubated at either 25°C or 30°C for 2 days.
