# Supplementary material for: Identification of factors that promote biogenesis of tRNACGASer
Source: RNA Biol. 2018 Oct 18;15(10):1286–94. doi: 10.1080/15476286.2018.1526539 (PMC6284589; doi:10.1080/15476286.2018.1526539)
Supplement: Supplemental Material [file krnb-15-10-1526539-s001.zip › Supplementary material/Supplementary Figure S3.pdf]

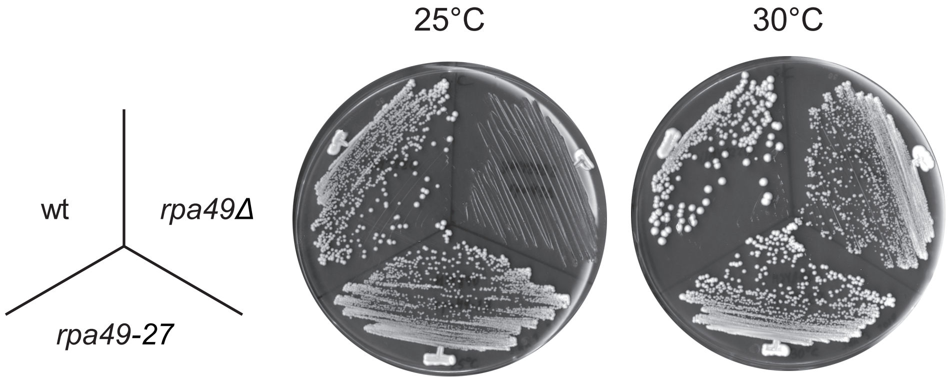

Figure S3. Growth phenotypes of strains with different *rpa49* alleles. The wild-type (UMY2220), *rpa49Δ* (UMY2902), and *rpa49-27* (MJY890) strains were streaked on SC plates and incubated at 25°C or 30°C for 3 days.
