# Supplementary material for: Identification of factors that promote biogenesis of tRNACGASer
Source: RNA Biol. 2018 Oct 18;15(10):1286–94. doi: 10.1080/15476286.2018.1526539 (PMC6284589; doi:10.1080/15476286.2018.1526539)
Supplement: Supplemental Material [file krnb-15-10-1526539-s001.zip › Supplementary material/Supplementary Figure S4.pdf]

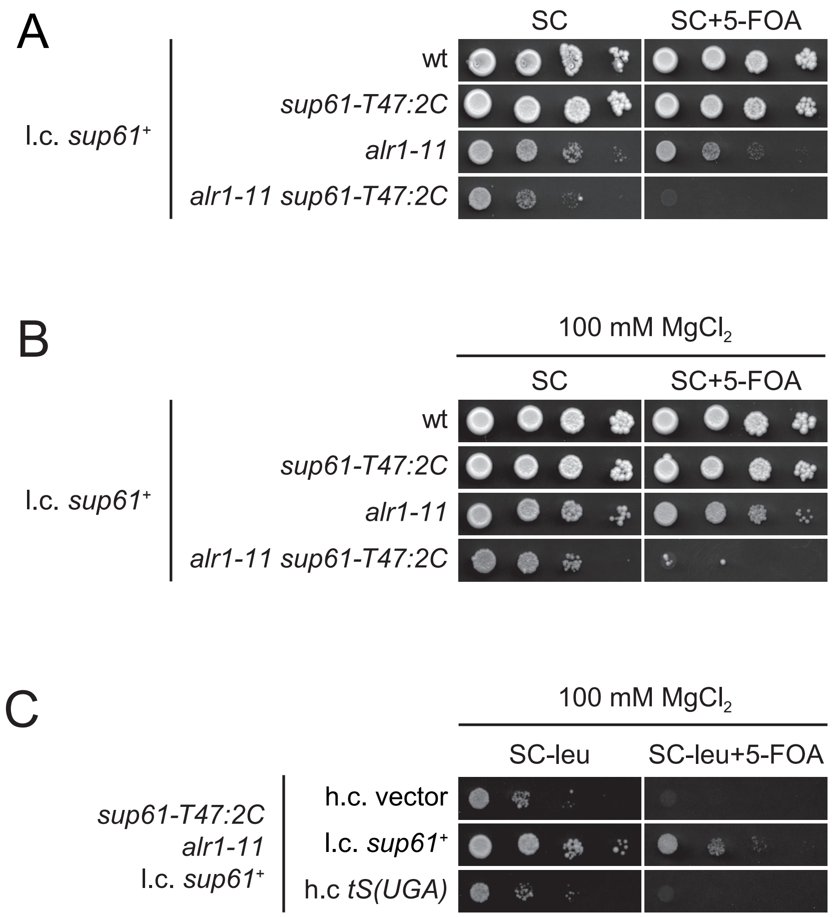

Figure S4. Growth phenotypes of strains with an *alr1-11* and/or *sup61-T47:2C* allele at 25°C. (A and B) The wild-type (UMY2220), *sup61-T47:2C* (MJY926), *alr1-11* (UMY3002), *sup61-T47:2C alr1-11* (UMY2967) strains carrying the l.c. *URA3* plasmid pMJ1421 (*sup61*+) were grown at 25°C in SC (A) or SC medium supplemented with 100 mM $MgCl_2$ (B). The cells were serially diluted, spotted onto SC and SC+5-FOA plates (A) or the same media supplemented with 100 mM $MgCl_2$ (B). The plates were incubated at 25°C for 3 days. (C) The *sup61-T47:2C alr1-11* strain described above was transformed with the indicated h.c. or l.c. *LEU2* plasmids. Cells were grown at 25°C in SC-leu medium supplemented with 100 mM $MgCl_2$, serially diluted, spotted onto SC-leu and SC-leu+5-FOA plates containing 100 mM $MgCl_2$, and incubated at 25°C for 3 days.
