# Supplementary material for: Identification of factors that promote biogenesis of tRNACGASer
Source: RNA Biol. 2018 Oct 18;15(10):1286–94. doi: 10.1080/15476286.2018.1526539 (PMC6284589; doi:10.1080/15476286.2018.1526539)
Supplement: Supplemental Material [file krnb-15-10-1526539-s001.zip › Supplementary material/Supplementary Table S1.docx]

**Table S1.** Relative abundance of various $\text{tRNA}_{\text{CGA}}^{\text{Ser}}$ species in indicated strains

| Growth temperature | Strain | Relative tRNA abundance^a^ | | | | |
| --- | --- | --- | --- | --- | --- | --- |
|  |  | $\text{tRNA}_{\text{CGA}}^{\text{Ser}}$ | pre-$\text{tRNA}_{\text{CGA}}^{\text{Ser}}$ | | | |
|  |  |  | Primary transcript | 5´-processed | End-processed | Total |
| 25°C | wt | 1.00 | 1.00 | 1.00 | 1.00 | 1.00 |
|  | *sup61-T47:2C* | 1.25 ± 0.22 | 1.23 ± 0.13 | 0.86 ± 0.12 | 1.41 ± 0.23 | 1.25 ± 0.19 |
|  | *ses1-40* | 1.04 ± 0.31 | 0.87 ± 0.10 | 0.77 ± 0.04 | 0.91 ± 0.11 | 0.87 ± 0.09 |
|  | *sup61-T47:2C ses1-40* | 0.43 ± 0.14 | 0.82 ± 0.11 | 0.38 ± 0.12 | 0.69 ± 0.10 | 0.66 ± 0.10 |
|  | *los1Δ* | 0.93 ± 0.09 | 0.92 ± 0.23 | 1.22 ± 0.47 | 2.83 ± 0.97 | 1.83 ± 0.56 |
|  | *sup61-T47:2C los1Δ* | 0.59 ± 0.05 | 1.01 ± 0.20 | 0.72 ± 0.06 | 1.84 ± 0.45 | 1.32 ± 0.26 |
| 25°C | wt | 1.00 | 1.00 | 1.00 | 1.00 | 1.00 |
|  | *sup61-T47:2C* | 1.21 ± 0.37 | 1.22 ± 0.15 | 0.85 ± 0.32 | 1.28 ± 0.40 | 1.15 ± 0.34 |
|  | *mot1-190* | 0.88 ± 0.18 | 0.93 ± 0.16 | 0.72 ± 0.16 | 0.90 ± 0.13 | 0.85 ± 0.04 |
|  | *sup61-T47:2C mot1-190* | 0.75 ± 0.13 | 0.90 ± 0.24 | 0.70 ± 0.32 | 1.12 ± 0.43 | 0.95 ± 0.34 |
| 30°C | wt | 1.00 | 1.00 | 1.00 | 1.00 | 1.00 |
|  | *sup61-T47:2C* | 0.87 ± 0.03 | 1.18 ± 0.06 | 0.60 ± 0.04 | 0.96 ± 0.07 | 0.90 ± 0.06 |
|  | *rpa49-27* | 0.80 ± 0.07 | 0.97 ± 0.05 | 0.77 ± 0.07 | 0.99 ± 0.14 | 0.93 ± 0.04 |
|  | *sup61-T47:2C rpa49-27* | 0.20 ± 0.11 | 1.09 ± 0.17 | 0.25 ± 0.07 | 0.44 ± 0.19 | 0.51 ± 0.15 |
|  | *rrn3-32* | 0.80 ± 0.13 | 1.14 ± 0.23 | 0.75 ± 0.20 | 0.95 ± 0.23 | 0.91 ± 0.21 |
|  | *sup61-T47:2C rrn3-32* | 0.31 ± 0.13 | 1.12 ± 0.33 | 0.41 ± 0.12 | 0.53 ± 0.20 | 0.61 ± 0.19 |

^a^ The signal for the indicated tRNA species was normalized to the corresponding $\text{tRNA}_{\text{i}}^{\text{Met}}$ signal and the value for each strain expressed relative to that for the wild-type. The total level of pre-$\text{tRNA}_{\text{CGA}}^{\text{Ser}}$ is the combined signal of the three precursor forms normalized to $\text{tRNA}_{\text{i}}^{\text{Met}}$. The values are derived from the blots shown in Fig. 2B, 3B, 3D and 2-3 additional independent experiments for each set of strains (see Fig. 2 and 3 for details). The standard deviation is indicated.
