# Supplementary material for: Identification of factors that promote biogenesis of tRNACGASer
Source: RNA Biol. 2018 Oct 18;15(10):1286–94. doi: 10.1080/15476286.2018.1526539 (PMC6284589; doi:10.1080/15476286.2018.1526539)
Supplement: Supplemental Material [file krnb-15-10-1526539-s001.zip › Supplementary material/Supplementary Table S2.docx]

Table S2. Relative abundance of modified nucleosides in the indicated strains.

| Strain | Nucleoside^a^ | | | | | | | | | |
| --- | --- | --- | --- | --- | --- | --- | --- | --- | --- | --- |
|  | D^b^ | Ψ | m^3^C | m^1^A+m^5^C | m^5^U | Um | Gm | ac^4^C | $\text{m}_{\text{2}}^{\text{2}}$G | i^6^A |
| wt | 1.00 | 1.00 | 1.00 | 1.00 | 1.00 | 1.00 | 1.00 | 1.00 | 1.00 | 1.00 |
| *ses1-40* | 0.88 ± 0.05 | 0.86 ± 0.08 | 0.99 ± 0.03 | 0.91 ± 0.04 | 0.92 ± 0.03 | 0.90 ± 0.13 | 0.89 ± 0.02 | 0.80 ± 0.21 | 0.88 ± 0.07 | 0.95 ± 0.02 |
| *los1∆* | 0.98 ± 0.02 | 0.97 ± 0.05 | 1.07 ± 0.14 | 1.05 ± 0.03 | 1.02 ± 0.03 | 0.88 ± 0.20 | 1.02 ± 0.01 | 0.96 ± 0.24 | 0.98 ± 0.03 | 0.98 ± 0.05 |
| *mot1-190* | 1.07 ± 0.12 | 1.05 ± 0.13 | 1 .20 ± 0.34 | 1.22 ± 0.24 | 1.08 ± 0.09 | 1.07 ± 0.11 | 1.16 ± 0.25 | 1.14 ± 0.23 | 1.10 ± 0.14 | 1.02 ± 0.08 |
| *rpa49-27* | 1.08 ± 0.06 | 1.05 ± 0.04 | 1.01 ± 0.08 | 1.23 ± 0.17 | 1.14 ± 0.07 | 1.02 ± 0.15 | 1.20 ± 0.12 | 1.10 ± 0.09 | 1.10 ± 0.06 | 1.06 ± 0.06 |
| *rrn3-32* | 0.99 ± 0.05 | 0.95 ± 0.07 | 0.89 ± 0.27 | 1.09 ± 0.11 | 1.04 ± 0.03 | 1.03 ± 0.17 | 1.08 ± 0.10 | 0.85 ± 0.16 | 0.99 ± 0.07 | 0.97 ± 0.06 |
| *alr1-11* | 0.97 ± 0.02 | 0.95 ± 0.06 | 0.98 ± 0.21 | 1.12 ± 0.11 | 1.09 ± 0.08 | 0.97 ± 0.25 | 1.02 ± 0.10 | 0.78 ± 0.19 | 0.99 ± 0.04 | 0.97 ± 0.03 |

Abbreviations: dihydrouridine (D); pseudouridine (Ψ); 3-methylcytidine (m^3^C); 1-methyladenosine (m^1^A); 5-methylcytidine (m^5^C); 5-methyluridine (m^5^U); 2’-*O*-methyluridine (U_m_); 2’-*O*-methylguanosine (G_m_); (ac^4^C) *N*^4^-acetylcytidine; *N*^2^,*N*^2^-dimethylguanosine ($\text{m}_{\text{2}}^{\text{2}}$G); *N*^6^-isopentenyladenosine (i^6^A)

^a^ The peak for the indicated nucleoside was integrated and the value normalized to the corresponding value for *N*^6^-threonylcarbamoyladenosine (t^6^A), which is not present in $\text{tRNA}_{\text{CGA}}^{\text{Ser}}$. As m^3^C co-elutes with U under our standard HPLC conditions, the normalized values for m^3^C are from re-analyses of the tRNA samples using a slightly modified elution buffer (see Materials and Methods). The normalized value for a nucleoside is expressed relative to the corresponding value in wild type cells which is set to 1. The values represent the average of three biological replicates and their standard deviation.

^b^ Dihydrouridine was detected at 215 nm.
