# Supplementary material for: Identification of factors that promote biogenesis of tRNACGASer
Source: RNA Biol. 2018 Oct 18;15(10):1286–94. doi: 10.1080/15476286.2018.1526539 (PMC6284589; doi:10.1080/15476286.2018.1526539)
Supplement: Supplemental Material [file krnb-15-10-1526539-s001.zip › Supplementary material/Supplementary Table S3.docx]

**Table S3.** *S. cerevisiae* strains used in this study.

| Yeast strain | Genotype | Source or  reference |
| --- | --- | --- |
| UMY2219 | *MATa ura3-1 leu2-3,112 trp1-1 his3-11,15 can1-100 ade2-1 ade3::hisG* | [[11](#_ENREF_11)] |
| UMY2220 | *MATα ura3-1 leu2-3,112 trp1-1 his3-11,15 can1-100 ade2-1 ade3::hisG* | [[11](#_ENREF_11)] |
| UMY2366 | *MATa/MATα ura3-1/ura3-1 leu2-3,112/leu2-3,112 trp1-1/trp1-1 his3-11,15/his3-11,15 can1-100/can1-100 ade2-1/ade2-1 ade3::hisG/ade3::hisG* | [[11](#_ENREF_11)] |
| UMY2256 | *MATa ura3-1 leu2-3,112 trp1-1 his3-11,15 can1-100 ade2-1 ade3::hisG sup61-T47:2C* | [[5](#_ENREF_5)] |
| MJY926 | *MATα ura3-1 leu2-3,112 trp1-1 his3-11,15 can1-100 ade2-1 ade3::hisG sup61-T47:2C* | This study |
| UMY2704 | *MATα ura3-1 leu2-3,112 trp1-1 his3-11,15 can1-100 ade2-1 ade3::hisG sup61-T47:2C* pMJ1421 | [[11](#_ENREF_11)] |
| UMY2872 | *MATα ura3-1 leu2-3,112 trp1-1 his3-11,15 can1-100 ade2-1 ade3::hisG dus2::kanMX4* | This study |
| UMY4286 | *MATα ura3-1 leu2-3,112 trp1-1 his3-11,15 can1-100 ade2-1 ade3::hisG sup61-T47:2C dus2::kanMX4* pRS316-*sup61^+^* | This study |
| UMY2565 | *MATα ura3-1 leu2-3,112 trp1-1 his3-11,15 can1-100 ade2-1 ade3::hisG mod5::TRP1* | This study |
| UMY4285 | *MATα ura3-1 leu2-3,112 trp1-1 his3-11,15 can1-100 ade2-1 ade3::hisG sup61-T47:2C mod5::TRP1* pRS316-*sup61^+^* |  |
| MJY924 | *MATα ura3-1 leu2-3,112 trp1-1 his3-11,15 can1-100 ade2-1 ade3::hisG ses1-40* | This study |
| MJY925 | *MATα ura3-1 leu2-3,112 trp1-1 his3-11,15 can1-100 ade2-1 ade3::hisG sup61-T47:2C ses1-40* | This study |
| UMY2441 | *MATa ura3-1 leu2-3,112 trp1-1 his3-11,15 can1-100 ade2-1 ade3::hisG los1::TRP1* | A. Esberg |
| UMY2861 | *MATa ura3-1 leu2-3,112 trp1-1 his3-11,15 can1-100 ade2-1 ade3::hisG sup61-T47:2C los1::TRP1* | This study |
| MJY1110 | *MATα ura3-1 leu2-3,112 trp1-1 his3-11,15 can1-100 ade2-1 ade3::hisG trm140::kanMX6* | This study |
| MJY1134 | *MATα ura3-1 leu2-3,112 trp1-1 his3-11,15 can1-100 ade2-1 ade3::hisG sup61-T47:2C trm140::kanMX6* | This study |
| MJY892 | *MATα ura3-1 leu2-3,112 trp1-1 his3-11,15 can1-100 ade2-1 ade3::hisG mot1-190* | This study |
| MJY893 | *MATα ura3-1 leu2-3,112 trp1-1 his3-11,15 can1-100 ade2-1 ade3::hisG sup61-T47:2C mot1-190* | This study |
| MJY890 | *MATα ura3-1 leu2-3,112 trp1-1 his3-11,15 can1-100 ade2-1 ade3::hisG rpa49-27* | This study |
| MJY891 | *MATα ura3-1 leu2-3,112 trp1-1 his3-11,15 can1-100 ade2-1 ade3::hisG* *sup61-T47:2C rpa49-27* | This study |
| MJY1111 | *MATα ura3-1 leu2-3,112 trp1-1 his3-11,15 can1-100 ade2-1 ade3::hisG rpa49::kanMX4* | This study |
| MJY894 | *MATα ura3-1 leu2-3,112 trp1-1 his3-11,15 can1-100 ade2-1 ade3::hisG rrn3-32* | This study |
| MJY895 | *MATα ura3-1 leu2-3,112 trp1-1 his3-11,15 can1-100 ade2-1 ade3::hisG sup61-T47:2C rrn3-32* | This study |
| UMY3002 | *MATα ura3-1 leu2-3,112 trp1-1 his3-11,15 can1-100 ade2-1 ade3::hisG alr1-11* | This study |
| UMY2967 | *MATα ura3-1 leu2-3,112 trp1-1 his3-11,15 can1-100 ade2-1 ade3::hisG sup61-T47:2C alr1-11* pMJ1421 | This study |
